# The parathyroid hormone-related protein receptor is expressed in breast cancer bone metastases and promotes autocrine proliferation in breast carcinoma cells

**DOI:** 10.1038/sj.bjc.6600757

**Published:** 2003-02-18

**Authors:** R P Hoey, C Sanderson, J Iddon, G Brady, N J Bundred, N G Anderson

**Affiliations:** 1 Division of Cancer Studies, School of Medicine; 2 School of Biological Sciences, University of Manchester, Oxford Road, Manchester M13 9PT, UK

**Keywords:** PTHRP, autocrine, mitogenesis, GPCR, MCF-7

## Abstract

Overproduction of parathyroid hormone-related protein (PTHRP) occurs in a high proportion of primary breast cancers (PBC) and is strongly implicated in their metastatic spread to bone. Although the PTHRP-receptor (PTHRP-R) is often coexpressed with PTHRP in PBC, its role in regulating breast cancer cell proliferation and metastases to bone remains unclear. The aims of this study were to determine the expression of the PTHRP-R in breast cancer bone metastases (BM) and to investigate the effects of PTHRP-R overexpression on breast cancer cell proliferation. PTHRP-R expression occurred in 85% (11 out of 13) of BM compared with 58% (39 out of 67) of PBC. Median expression was higher (*P*<0.05) in BM compared with PBC. PTHRP increased cAMP accumulation and DNA synthesis in MCF-7 cells stably overexpressing the PTHRP-R (MCF-7^WTR^) but not in MCF-7^VEC^ control cells. The increase in DNA synthesis was mimicked by the cAMP pathway activator forskolin. The receptor antagonist PTHRP_7–34_ reduced DNA synthesis in MCF-7^WTR^ cells, but not MCF-7^VEC^ cells, indicating that receptor overexpression promotes autocrine PTHRP activity. MCF-7^WTR^ cells showed increased mitogenic responsiveness to fetal calf serum and reduced doubling times. PTHRP induced weak activation of ERK1 and ERK2 and potentiated their activation by serum growth factors. Collectively these results show that the PTHRP-R is frequently expressed in breast cancer BM and indicate that receptor overexpression drives proliferation via autocrine signals that are mediated via cAMP and ERK pathways.

Parathyroid hormone-related protein (PTHRP) was first identified as the causative factor in the paraneoplastic syndrome humoral hypercalcaemia of malignancy (HHM) (Suva *et al*, 1987). The amino-terminal region of PTHRP is similar to that in parathyroid hormone (PTH) and both hormones share a common G protein-coupled receptor, known as the PTH/PTHRP receptor (PTHRP-R). In contrast to PTH, which is produced almost exclusively by the parathyroid gland and circulates as an endocrine regulator of calcium homeostasis, PTHRP has been found in almost every tissue and acts via paracrine or autocrine mechanisms to regulate development and cell growth and differentiation (reviewed in Wysolmerski and Stewart, 1998).

Substantial evidence indicates that PTHRP is involved in breast cancer progression. In particular, a number of studies strongly implicate PTHRP as having a major role in the preferential metastatic spread of breast cancers to the skeleton. For example, compared to primary breast tumours and non-skeletal metastases, PTHRP is more frequently expressed in bone metastases (BM) (Powell *et al*, 1991). Moreover, expression of PTHRP in primary tumours predicts the subsequent development of BM (Bundred *et al*, 1992; Bouizar *et al*, 1993) and correlates with poor prognosis (Yoshida *et al*, 2000). Tumour cells secreting PTHRP may be better able to survive and proliferate in bone since localised production of PTHRP by breast cancer cells in these sites has been shown to promote osteolysis in a mouse model of skeletal metastasis (Guise *et al*, 1996).

In contrast to the substantial body of evidence linking PTHRP to breast cancer progression, much less is known about the role played by the PTHRP-R. Several breast tumour cell lines express the PTHRP-R and proliferate in response to PTHRP (Birch *et al*, 1995; Cataisson *et al*, 2000). Other studies have detected PTHRP-R expression in primary tumours (Carron *et al*, 1997; Downey *et al*, 1997; Iezzoni *et al*, 1998). Moreover, PTHRP-R and ligand are coexpressed in majority of these tumours supporting the idea that there may be paracrine or autocrine mechanisms involving PTHRP that are important for tumour progression. In support of this, we recently found that coexpression of PTHRP-R and ligand in primary breast cancers predicts poor patient prognosis (Linforth *et al*, 2002).

Overexpression of PTHRP and its receptor in breast tumour cells could also promote the growth of such cells in skeletal metastases by stimulating their proliferation in an autocrine fashion. To investigate this idea, we have set out to establish whether breast cancer cells maintain or upregulate PTHRP-R expression in these lesions by comparing the frequency and extent of PTHRP-R expression in BM with that in primary breast cancers. In consequence of our findings, we then characterised how overexpression of the PTHRP-R in breast cancer cells influences their proliferative responsiveness.

## MATERIALS AND METHODS

### Assessment of PTHRP-R expression in primary breast cancers and skeletal metastases

Following ethical approval, samples of malignant breast carcinoma tissue and BM were collected from patients undergoing surgery at the South Manchester University Hospital. After surgical excision, samples were removed from the tumour and snap frozen in liquid nitrogen. Bone metastases were collected during surgery on patients presenting with acute pathological fractured femurs because of breast cancer, who underwent open reduction and internal fixation of the fracture.

RNA was extracted from tissue samples using Trizol™ (Invitrogen, Paisley, UK) in accordance with the manufacturer's protocol. RNA yield was quantitated by UV spectrophotometry and the integrity verified on agarose gels. PCR analysis of PTHRP-R expression was carried out following global amplification of expressed genes by poly(A) PCR (Brady and Iscove, 1993; Al-Taher *et al*, 2000). Direct comparison of mRNA expression levels measured using this technique gave identical results to those obtained using conventional RT–PCR or TaqMan real-time quantitative PCR (Brady, unpublished data). For gene-specific PCR, reactions, were carried out in a total volume of 22 *μ*l and contained 1 ng of poly(A) cDNA, 0.33 *μ*M each oligonucleotide PCR primer, 0.5 U Taq polymerase (Roche Biochemicals, Lewes, UK) and 0.25 mM dNTPs. PCR primers for the PTHRP-R were directed towards the mRNA sequence within 300 bp of the poly(A) addition site, as described previously (Al-Taher *et al*, 2000): forward primer (1734–1756) CCG CCT ACT GCC CAC TGC CAC CAC, reverse primer (1973–1996) TCC ATC CAC TAT GTC AGC AGG TCC. The reactions were carried out in a programmable thermocycler (Techne PHC-3) using the following conditions: one cycle of 2 min at 94°C, then 25–35 cycles of 30 s at 94°C, 30 s at 60°C and 1 min at 72°C, and finally one cycle of 5 min at 72°C.

In order to quantify gene expression, PCR reaction products were run on a 1% agarose gel alongside a series of standards containing known amounts of PCR product DNA. These were prepared by combining the gene-specific PCR products generated from several reactions and diluting these to produce a series containing a known amount of DNA molecules from 1.5×10^10^ to 1.5×10^4^ ml^−1^, as described (Al-Taher *et al*, 2000). Dilutions were made using a solution of sonicated carrier lambda DNA.

Following agarose gel electrophoresis, gels were denatured in 1.5 M NaCl, 0.5 M NaOH for 45 min and then neutralised with 1 M NH_4_OH before blotting on to Hybond-N membrane (Amersham, Bucks, UK). The membrane was then rinsed in sodium phosphate buffer and prehybridized for 2 h at 55°C with denatured salmon sperm DNA. Membranes were then hybridised at 55°C overnight with a ^32^P-labelled oligonucleotide probe directed to a specific sequence within the PTHRP-R PCR product, as follows: (1852–1875) GAC GAT GGG TTC CTC AAC GGC TCC. The membrane was then washed, dried and exposed to X-ray film for 1–3 days. In addition, the hybridised bands were quantified using a phosphorimager. The values for the DNA standards were used to construct a standard curve from which values for the samples were read. Samples falling out with the linear range of the standard curve were resubjected to PCR using a modified number of amplification cycles.

### PTHRP-R cDNA constructs and generation of stable transfected cell populations

Wild-type human PTHRP-R cDNA in pcDNA1 plasmid vectors were generously provided by Dr E Schipani (Boston). The receptor cDNA was excised and subcloned into pCIneo (Promega, Southampton, UK) vector. Insertion was confirmed by restriction digestions using *Eco*R1 and *Xba*I, and by direct sequencing. To generate cell lines stably expressing the PTHRP-R, MCF-7 cells (obtained from the ATCC and grown in DMEM containing 10% fetal calf serum (FCS) and 2 mM glutamine) were transfected with 9 *μ*g of the receptor cDNA or with empty pCIneo vector as a control using FuGene6 (Roche Biochemicals) according to the manufacturer's instructions. Following transfection, cells were placed in a selection medium containing 0.5 mg ml^−1^ G418 (Invitrogen). After approximately 2 weeks, during which the medium was changed every 2 days, resistant cells were expanded for analysis. Subcloning of transfected cells was not performed and all of the experiments reported in this study were carried out using the entire population of G418-resistant cells.

### PTHRP-R expression by RT–PCR in transfected stable lines

Total RNA was extracted from approximately 5×10^6^ cells using the RNeasy kit (Qiagen, Crawley, UK). RT–PCR was carried out using the enhanced avian RT–PCR kit (Sigma) with the following primers designed to amplify a 571 bp fragment:

forward 5′-AGGAACAGATCTTCCTGCTGCA-3′;

reverse 5′-TGCATGTGGATGTAGTTGCGCGT-3′.

The reactions used 100 ng RNA with primers at a final concentration of 250 nM in the presence of 3 mM MgCl_2_. First strand synthesis was conducted at 42°C for 50 min, followed by denaturation at 94°C for 2 min. A total of 35 cycles of denaturation (94°C, 15 s) and annealing (68°C, 1 min) were followed by extension at 68°C for 5 min. Reaction products were run alongside 100 bp size markers on 1% agarose gels containing ethidium bromide and the resulting gel photographed under UV light.

### Cyclic AMP (CAMP) assay

This was carried out using the cAMP binding protein competitive assay as described previously (Yarwood *et al*, 1998). In brief, cells in six-well plates were starved overnight in serum-free medium prior to stimulation with factors as described. Perchloric acid extracts were then prepared followed by neutralisation and removal of precipitated material by centrifugation. Supernatants were stored at −80°C prior to the assay. Samples were assayed in the presence of 0.1 *μ*Ci [^3^H]cAMP and 8 *μ*g cAMP binding protein (Sigma) alongside a series of standard cAMP solutions. Following binding to equilibrium (2–3 h), unbound cAMP was precipitated by the addition of activated charcoal suspension. The suspension was centrifuged and an aliquot of the supernatant solution counted on a scintillation counter. Results are expressed as picomoles of cAMP formed per million cells.

### Thymidine uptake assay

Cells were grown to approximately 80% confluence in 24-well plates. Cells were starved for 24 h in serum-free medium and then test agents were added in fresh serum-free medium for a further 24 h. In experiments testing the effects of PTHRP_7–34_, following incubation in serum-free medium, cells were treated for a further 24 h in fresh serum-free medium or in a medium containing 0.5% serum. Cultures received additionally either drug vehicle (0.0001% acetic acid) or PTHRP_7–34_ (1 *μ*M). During the final 4 h 0.25 *μ*Ci [^3^H]thymidine (ICN) was added. Cells were then washed three times with PBS followed by the addition of 0.5 ml ice-cold TCA (20% w v^−1^) for 1 h. The cell precipitates were washed twice with 20% TCA, once with 95% ethanol, then dissolved in 0.25 ml 1 M NaOH and neutralised with an equal volume of 1 M HCl. Aliquots were counted on a scintillation counter.

### Measurement of doubling time

Cells were plated in quadruplicate at low density in a medium containing 2% serum. Cell numbers were counted using a haemocytometer every 24 h for a total of 96 h. The average time taken for each cell population to double in number was then calculated from the logarithmic phase of the growth curve.

### Measurement of PTHRP secretion

PTHRP was measured using a two-site immunoradiometric assay (IDS Ltd, Tyne and Wear, UK). Cells were plated in triplicate (10 000 cells well^−1^) in a 48-well plate. After 72 h, the medium was changed to serum-free medium and the cells were incubated for 48 h. The conditioned medium was then collected and immediately assayed alongside a series of PTHRP standards (0–40 pmol). To allow for variations in growth rate (see Results), cells were removed and counted. PTHRP accumulation was expressed as pico moles produced by 10^6^ cells over the 48 h incubation period.

### ERK activation

ERK activation was assessed by immunoblotting cell lysates with antiphospho-ERK antibodies as described previously (Yarwood *et al*, 1999). Briefly, cells were starved in serum-free medium overnight and then stimulated in fresh medium with either EGF, IGF-I or PTHRP_1–34_ at the concentrations and for the times detailed in the figure legends. Lysates were prepared and equivalent amounts of cellular protein (20 *μ*g) were run out on 12% SDS–PAGE gels. The samples were transferred to Hybond ECL (Amersham Bioscience, Bucks, UK), which was then blocked and probed with antiphospho-ERK antibodies (Cell Signalling Technology, Hitchin, UK) at 1 : 2000 dilution. Immunoreactive bands were visualised following HRP-linked secondary antibody incubation and reaction of the blots with Supersignal West Pico (Pierce, Warrington, UK). Identical samples were run in parallel and immunoblotted with anti-ERK total antibodies (Santa Cruz, CA, USA) to confirm equivalent sample loading.

## RESULTS

**Figure 1:**
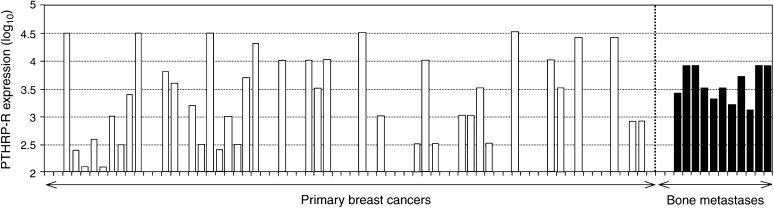
Expression of the PTHRP-R in primary breast cancers and BM. PTHRP-R expression was measured in 67 primary breast cancers and 13 bone metastases by RT–PCR as described in Materials and Methods. Relative expression was quantified by Southern blotting of the agarose gels using a specific ^32^P-labelled probe followed by phosphorimaging, and related to a series of standards of known amounts of DNA corresponding to the PCR product. Owing to the wide variation in receptor expression within the primary cancer samples data were log_10_ transformed for clarity.

In order to investigate the potential importance of PTHRP-mediated autocrine actions in metastatic breast cancer cells, we initially examined whether the PTHRP-R was expressed in skeletal metastases. Expression was measured by semiquantitative RT–PCR followed by Southern blotting and scanning densitometry. The data, presented in Figure 1

, show that the PTHRP-R is detectable in around 58% (39 of 67) of the primary breast cancers compared to 85% (11 of 13) of the BM. While this difference just fails to reach statistical significance (*P*=0.053, *χ*^2^ test), these results indicate that the PTHRP-R is expressed more frequently in BM. In addition, although expression levels varied considerably, especially among the primary cancer samples, the median receptor expression level was higher in the metastases samples compared to the primary cancers (2818 (1189–7483) *vs* 299 (10–3000); median and interquartile range (*P*<0.05; Mann–Whitney *U*-test)) (Figure 1).

**Figure 2:**
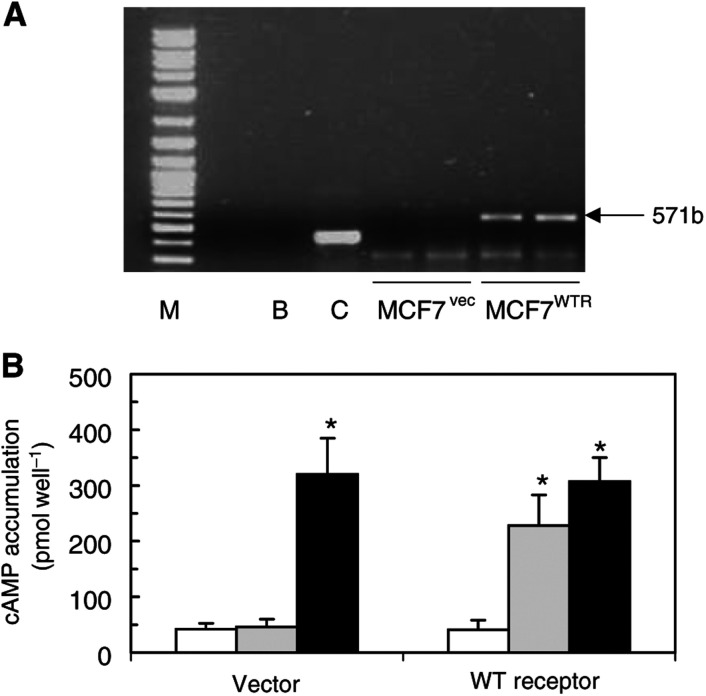
Comparison of PTHRP-R expression and responsiveness to PTHRP in vector- and receptor-transfected MCF-7 cells. (**A**) Total RNA was isolated from MCF-7^VEC^ and MCF-7^WTR^ cells, and PTHRP-R expression was measured by RT–PCR as described in Materials and Methods. The specific receptor fragment of 571 bp is indicated. M=100 bp size markers, B=water blank reaction without RNA, C=control reaction containing irrelevant RNA and primers. (**B**) Cells in six-well culture plates were pretreated for 10 min with IBMX (0.5 mM) followed by treatment for a further 10 min with vehicle (ethanol, 0.01%; open bars), PTHRP (125 nM; grey bars) or forskolin (1 *μ*M; black bars). Extracts were then prepared and assayed for cAMP content as described in Materials and Methods. The data represent means±s.e.m. from three independent experiments for each cell population. Individual assays were performed in triplicate. ^*^Values significantly different from the corresponding control value (*P*<0.05; ANOVA).

Expression of the PTHRP-R in metastatic breast cancers supports the idea that tumour cells within these lesions are responsive to PTHRP. Based on previous reports, showing that PTHRP can increase proliferation in some breast cancer cell lines, we reasoned that overexpression of the receptor would increase mitogenic responses of cells to PTHRP. To examine this, MCF-7 breast carcinoma cells were transfected with a cDNA encoding the human PTHRP-R. Following transfection and selection, we confirmed stable receptor expression by RT–PCR and by measuring the response of these cells to PTHRP in terms of cAMP generation. Stable populations of cells transfected with PTHRP-R cDNA (MCF-7^WTR^) expressed the receptor, whereas vector-transfected cells (MCF-7^VEC^) did not (Figure 2A

). MCF-7^WTR^ cells exhibited a ≈five-fold increase in cAMP accumulation in response to PTHRP_1–34_, whereas MCF-7^VEC^ cells showed little or no responsiveness to PTHRP_1–34_ (Figure 2B). The response of both cell types to forskolin, which directly activates adenylyl cyclase, was virtually identical indicating that receptor transfection did not affect the basic functioning of the cAMP generation system.

**Figure 3:**
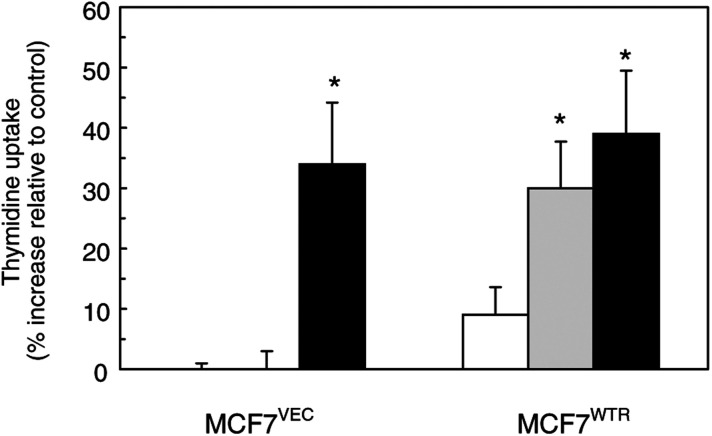
Effect of PTHRP_1–34_ on DNA synthesis in vector and PTHRP-R-transfected MCF-7 cells. Cells in 24-well culture dishes were incubated in serum-free medium for 24 h prior to treatment for a further 24 h with PTHRP_1–34_ (25 nM; open bars or 125 nM; grey bars) or forskolin (100 nM; black bars). [^3^H]thymidine (0.25 *μ*Ci well^−1^) was added for the final 4 h, then cellular uptake of thymidine was measured as described in Materials and Methods. The data represent the increase (%) in thymidine uptake relative to unstimulated cells for each cell population and are means±s.e.m. from three independent experiments for each line. Individual measurements were performed in triplicate. ^*^Values significantly different from respective control (*P*<0.05, ANOVA).

To investigate the effects of PTHRP-R overexpression on mitogenesis, we first examined the effect of exogenously added PTHRP on thymidine uptake, as a measure of cell cycle progression. MCF-7^VEC^ cells were unresponsive to PTHRP_1–34_ administered at two different concentrations (Figure 3

). By contrast, MCF-7^WTR^ cells showed a significant increase in thymidine uptake in response to PTHRP_1–34_ treatment. Exposure of both cell types to 100 nM forskolin resulted in quantitatively similar increases in thymidine incorporation indicating that cAMP signals a mitogenic response in these cells.

**Figure 4:**
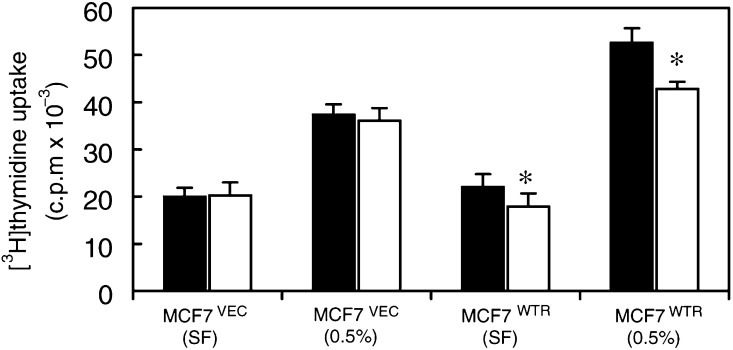
PTHRP_7–34_ inhibits DNA synthesis. Cells in 24-well culture dishes were incubated in serum-free medium for 24 h prior to treatment for a further 24 h in a serum-free conditions (SF) or in a medium containing 0.5% serum (0.5%). Cultures received additionally either drug vehicle (0.0001% acetic acid, black bars) or PTHRP_7–34_ (1 *μ*M, white bars). [^3^H]thymidine (0.25 *μ*Ci well^−1^) was added for the final 4 h, then cellular uptake of thymidine was measured as described in Materials and Methods. The data represent means±s.d. from a single experiment assayed in quadruplicate, which was repeated on another occasion with similar results. ^*^Values significantly different from PTHRP_7–34_ treatment (*P*<0.05, Student's *t*-test).

We next sought evidence for autocrine-mediated effects of PTHRP on proliferation. Previous studies have shown that MCF-7 cells synthesise PTHRP mRNA (Birch *et al*, 1995) and secrete PTHRP into the medium (Rong *et al*, 1999). To confirm these findings, we measured PTHRP secretion in each cell type using a sensitive two-site immunoradiometric assay. These assays revealed that PTHRP was present in the conditioned medium and that the degree of PTHRP production was similar in each cell type (MCF-7^VEC^: 1.67±0.05 pmol 10^4^ cells^−1^ 48 h^−1^; MCF-7^WTR^ cells: 1.58±0.06). Since MCF-7 cells produce PTHRP, overexpression of the PTHRP-R may sensitise such cells to autocrine activity. To test this further, we examined the effects of PTHRP_7–34_, which acts as an antagonist of the PTHRP-R and blocks ligand-induced receptor activation (Nagasaki *et al*, 1989). Figure 4

shows that incubation of MCF-7^WTR^ cells, in either serum-free medium or medium containing 0.5% serum, with PTHRP_7–34_ significantly reduced thymidine incorporation by 18 and 36%, respectively. In contrast, MCF-7^VEC^ cells were unaffected by treatment with PTHRP_7–34_. These experiments demonstrate that overexpression of the wild-type PTHRP-R alone, in the absence of any alterations in PTHRP ligand production, is sufficient to confer autocrine proliferative responsiveness to PTHRP.

We next examined whether overexpression of the PTHRP-R affected the general mitogenic responsiveness of MCF-7 cells. Exposure of previously starved cells to normal growth medium containing 10% FCS led to increases in thymidine uptake that were similar in MCF-7^VEC^ and MCF-7^WTR^ cells (data not shown). However, when the cells were treated instead with a medium containing 2% serum, significant differences in responsiveness were revealed. Stimulation of MCF-7^WTR^ cells resulted in a 122±9% increase in thymidine uptake compared with MCF-7^VEC^ cells where the increase was 78±9% (Table 1

**Table 1:** Effect of PTHRP-R expression on mitogenesis

**Cell population**	**2% Serum-stimulated thymidine uptake (%)**	**Doubling time (h)**
Vector	78±9	25.09±1.04
WT receptor	122±9*	20.48±1.69*

Cells were placed in serum-free medium for 24 h and then stimulated with a medium containing 2% serum for a further 24 h. [^3^H]thymidine uptake was measured as described in Materials and Methods. To measure doubling times, cells were plated in quadruplicate at low density in a medium containing 2% serum. Cell numbers were counted every 24 h for a total of 96 h. The average time taken for each cell population to double in number was then calculated from the logarithmic phase of the growth curve. The data are means±s.d. and are derived from three independent experiments.

* A significant difference between MCF-7^VEC^ and MCF-7^WTR^ cells (*P*<0.05, ANOVA and Student's *t*-test).

). Consistent with their apparent increased mitogenic responsiveness to serum, the MCF-7^WTR^ cells also exhibited a doubling time that was significantly lower than that measured for MCF-7^VEC^ cells (Table 1). These results suggest that overexpression of the PTHRP-R increases the capacity of MCF-7 cells to respond to mitogenic factors present in FCS.

The preceding data indicate that overexpression of the PTHRP-R increases the mitogenic responsiveness of breast cancer cells not only to endogenous and exogenous PTHRP but also to heterologous serum-derived growth factors. To begin to explore the intracellular mechanisms that might mediate these effects, we elected to characterise the signalling pathways that mediate the proliferative actions of PTHRP in MCF-7 cells. The PTHRP-R can couple to multiple signalling systems, including Ca^2+^/protein kinase C, cAMP and Ras/ERK pathways (Carpio *et al*, 2001; Miao *et al*, 2001). PTHRP did not induce Ca^2+^ accumulation in MCF-7 cells (data not shown) in agreement with a previous report (Birch *et al*, 1995), making it unlikely that this pathway contributes to the mitogenic actions of PTHRP. We attempted to address directly whether cAMP signals were required for the proliferative actions of PTHRP using H89, a selective inhibitor of cAMP-dependent protein kinase. However, at concentrations required to block PKA (5–10 *μ*M) this agent caused extensive cell detachment and apoptosis within a few hours, thus precluding its use in the mitogenesis assay.

**Figure 5:**
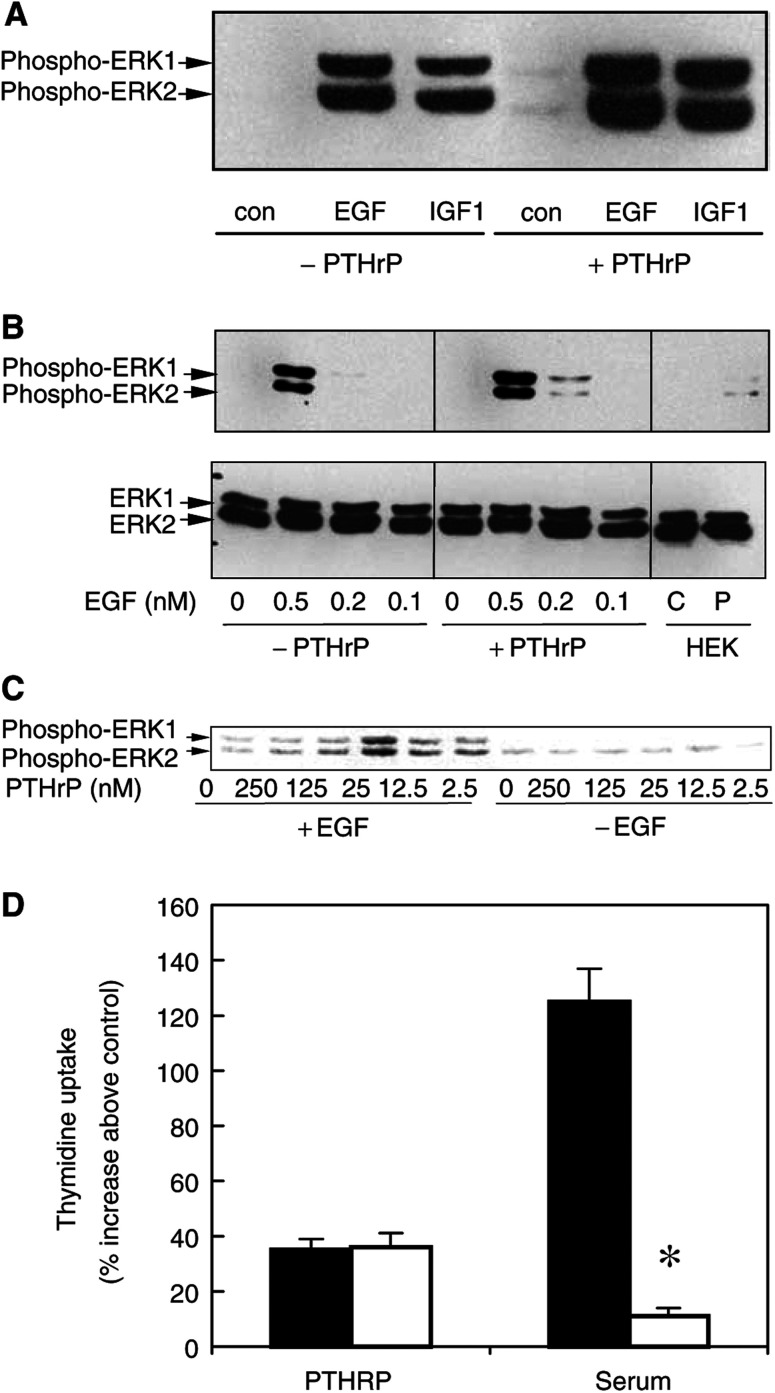
PTHRP_1–34_ potentiates both EGF- and IGF-1-induced activation of ERK MAP kinases. MCF-7^WTR^ cells were starved in serum-free medium overnight. (**A**) The cells were then treated for 5 min with PTHRP_1–34_ (125 nM) or vehicle followed by nothing, EGF (2 nM) or IGF-1 (2 nM) for a further 5 min. (**B**) Cells were pretreated with buffer (−PTHRP) or with PTHRP (125 nM) for 5 min prior to stimulation with the stated concentrations of EGF for a further 5 min. Lanes labelled C and P represent samples from control or PTHRP-treated HEK293 cells expressing PTHRP-R, run as a positive control. (**C**) Cells were treated with the stated concentrations of PTHRP_1–34_ for 5 min prior to further stimulation with 0.5 nM EGF for 5 min. Lysates were prepared and Western blotted for phospho-ERK1/2 (top panel) as described in Materials and Methods. The data are representative of experiments carried out on five separate occasions. (**D**) MCF7^WTR^ cells were incubated in serum-free medium for 24 h prior to treatment for a further 24 h with PTHRP_1–34_ (125 nM) or 2% serum in the absence (filled bars) or presence (open bars) of 25 *μ*M PD098059. [^3^H]thymidine uptake was then measured as described in Materials and Methods. The data represent the increase (%) in thymidine uptake relative to cells maintained in serum-free medium are means±s.e.m. from three independent experiments. ^*^Values significantly different from respective control (*P*<0.01, Student's paired *t*-test).

To examine whether the ERK pathway might play a role in the mitogenic actions of PTHRP, MCF-7^WTR^ cells were stimulated with PTHRP_1–34_ and, for comparison, EGF and IGF-1, two growth factors known to act as potent mitogens for MCF-7 cells (Pollak *et al*, 1988; Godden *et al*, 1992). ERK activation was assessed by immunoblotting with antibodies recognising the dually phosphorylated forms of ERK1 and ERK2, which accurately reflects their activation status (Anderson *et al*, 1990). As shown in Figure 5A

, PTHRP_1–34_ induced a small increase in the phosphorylation of both ERK1 and ERK2. The size of this increase was negligible compared to that induced by either EGF or IGF-1. However, when PTHRP was administered prior to EGF or IGF-1, there was a potentiation of the resultant ERK phosphorylation (Figure 5A). The degree of potentiation was dependent upon the dose of EGF administered. Use of suboptimal EGF concentrations revealed the potentiating action of PTHRP on EGF-induced ERK activation more clearly (Figure 5B). Conversely, potentiation of EGF-induced activation of ERKs was also dependent on the concentration of PTHRP administered (Figure 5C). These results indicate that PTHRP by itself is a weak activator of the ERK pathway in MCF-7 cells, but that it can boost ERK activation by other growth factors. We finally tested whether the ERK pathway was required for the proliferation of MCF-7 cells. Cells were incubated with PD098059, which blocks the activation of MEK, the protein kinase required for activation of ERKs (Alessi *et al*, 1995). As shown in Figure 5D, although blockade of the ERK pathway almost completely prevented the increase in [^3^H]thymidine induced by 2% serum, it had no effect on the ability of PTHRP to increase thymidine uptake. Thus, although PTHRP-induced mitogenesis may occur through an ERK-independent mechanism, the ability of PTHRP to potentiate the activation of ERK by EGF and IGF-1, together with the ability of cells to respond to autocrine PTHRP, could provide one explanation for the increased mitogenic responsiveness and reduced doubling times of MCF-7 cells overexpressing the PTHRP-R.

## DISCUSSION

PTHRP is now well recognised as an important factor in the development of osteolytic BM from breast cancer. The mechanisms involved remain to be fully resolved but studies by Guise and colleagues provide evidence that production of PTHRP forms part of a vicious cycle involving osteoclast activation followed by the release of mitogenic growth factors such as IGF-1 from the bone matrix (Guise, 2000). In this model, PTHRP exerts its actions by binding to receptors present on osteoblasts, which in response secrete factors leading to osteoclast activation. The presence or absence of the PTHRP-R on the tumour cells themselves is unlikely to exert a major influence in such a process. On the other hand, PTHRP-R expression may give metastatic breast cancer cells a selective advantage that allows them to colonise and expand in secondary sites by permitting autocrine PTHRP signalling. In this study, we demonstrate that PTHRP-R expression is indeed maintained in breast cancer BM. Indeed our data suggest that PTHRP-R expression occurs more frequently and at a higher level in BM compared to the primary cancer. This suggests that tumour cells overexpressing the PTHRP-R have a selective survival or growth advantage in BM. Alternatively, tumour cells may upregulate PTHRP-R expression as a result of their exposure to the bone microenvironment. Distinguishing between these possibilities is a goal for future studies.

The presence of the PTHRP-R in metastatic breast tumour cells clearly indicated that these cells could respond to PTHRP in a paracrine or autocrine manner. The results of our analyses of MCF-7 breast cancer cells engineered to express the receptor provide evidence that PTHRP-R overexpression may be sufficient to give cells a selective advantage in terms of their proliferative capacity. We show that this advantage may include not only an increased capacity to respond to both exogenous and endogenous PTHRP, but also to heterologous growth factors.

A previous report (Birch *et al*, 1995) showed that parental MCF-7 cells responded mitogenically to exogenously applied PTHRP. The reason why we failed to observe any such effect of PTHRP in our vector-transfected cells may be because of differences in the culture conditions used. Nevertheless, our work shows that mitogenic responsiveness to exogenous PTHRP increases when the receptor is overexpressed.

Our results also show that the proliferation of MCF-7 cells overexpressing the PTHRP-R is sustained in part by PTHRP acting in an autocrine manner. The antiproliferative effects of PTHRP-R antagonism were greater in cells growing in the presence of serum. Since PTHRP is not present at detectable levels in normal FCS (CS and NGA, unpublished data), this suggests that the proliferation of MCF-7 cells in serum is supported to a greater extent by autocrine PTHRP. This may be because growth factors present in serum stimulate the production of endogenous PTHRP. Interestingly, *PTHRP* gene expression is reported to be promoted via the Ras-ERK pathway (Aklilu *et al*, 2000), and many of the growth factors present in serum are well known to activate this pathway.

Apart from acting through the PTHRP-R, recent studies have uncovered an alternative mechanism through which PTHRP can exert cellular actions. This involves an intracrine pathway through which newly synthesised PTHRP is directed to the nucleus. The precise role that this novel pathway plays in controlling cellular function is still unclear, but it is interesting to note that the overexpression of PTHRP in MCF-7 cells results in increased proliferation via an intracrine mechanism (Falzon and Du, 2000). Intracrine PTHRP signalling required overexpression of PTHRP indicating that this pathway is unlikely to operate with the endogenous levels of PTHRP present in our cell system. Nevertheless, it would be interesting to test whether intracrine and autocrine PTHRP activity coexist in cells with overexpression of both PTHRP and the receptor.

In addition to conferring increased responsiveness to PTHRP, our results also show that overexpression of the PTHRP-R sensitises cells to the mitogenic actions of heterologous growth factors present in serum. One potential explanation for these results could involve, as suggested above, an increase in the synthesis of PTHRP in cells cultured in a serum-containing medium. Cells expressing the PTHRP-R would show increased autocrine responsiveness to PTHRP. Our results, showing that PTHRP-R antagonism is more effective in the presence of serum, support this conclusion.

An additional explanation for the apparent increased mitogenic responsiveness of cells expressing the PTHRP-R derives from the results of our analysis of ERK signalling. We found that PTHRP indirectly influences signalling through the ERK pathway; while PTHRP had little effect on its own, it increased the resulting activation of ERK when cells were stimulated by EGF and IGF-1. The mechanisms involved remain to be established, although it is interesting to note that ATP, which also signals via a GPCR, potentiated EGF signalling in MCF-7 cells (Wagstaff *et al*, 2000). Since the ERK pathway appears critical for optimal proliferation in MCF-7 cells (Hermanto *et al*, 2000), the potentiation of its activation by PTHRP may be significant and could provide a further explanation for the enhanced mitogenesis in cells overexpressing the PTHRP-R.

Finally, our studies provide some insight into the mechanisms by which PTHRP induces mitogenesis in MCF-7 cells. Although the ERK pathway is required for optimal proliferation in MCF-7 cells, blockade of its activation using the chemical inhibitor PD098059 failed to affect PTHRP-induced mitogenesis, ruling out any involvement of this pathway in the direct mitogenic effects of PTHRP.

As shown in many other systems, the PTHRP-R couples via G_s_ to adenylyl cyclase and the cAMP signalling pathway (Gardella and Juppner, 2001). Although we were unable to test directly whether this pathway mediates the mitogenic effects of PTHRP in MCF-7 cells, the fact that forskolin, a direct activator of adenylyl cyclase, also promoted mitogenesis indicates that cAMP is mitogenic in these cells. This appears to contradict previous reports suggesting that the cAMP signalling system inhibits proliferation in MCF-7 cells (Chen *et al*, 1998). We believe that this apparent difference may be explained by the relative strength of the cAMP signal generated. We have shown previously that fibroblasts can respond differentially to low/transient *vs* high/sustained levels of cAMP (Yarwood *et al*, 1998). In the present study, low concentrations of forskolin promoted mitogenesis whereas high concentrations were inhibitory (RP and NGA, unpublished data). The antiproliferative effects reported in the study by Chen *et al* resulted from the introduction of dominantly active version of G_s_ into the cells, and although they did not report the levels of cAMP generated by this procedure, it is likely that such a manipulation would lead to sustained cAMP signalling. We therefore contend that PTHRP promotes mitogenesis in MCF-7 cells by generating transient increases in cAMP.

To conclude, we have shown that the PTHRP-R is expressed in breast cancer BM, and thus may mediate autocrine PTHRP signalling in these lesions. Overexpression of the PTHRP-R in a breast cancer cell line increases the mitogenic responsiveness of these cells not only to PTHRP but also to heterologous growth factors. PTHRP can promote mitogenesis in an autocrine manner via receptor-mediated increases in cAMP and by sensitising the ERK signalling pathway to stimulation by other growth factors. Strategies aimed at blocking PTHRP-R activation may therefore slow the progression of breast cancer cells in BM.
